# Automatic segmentation of rectal tumor on diffusion‐weighted images by deep learning with U‐Net

**DOI:** 10.1002/acm2.13381

**Published:** 2021-08-03

**Authors:** Hai‐Tao Zhu, Xiao‐Yan Zhang, Yan‐Jie Shi, Xiao‐Ting Li, Ying‐Shi Sun

**Affiliations:** ^1^ Department of Radiology Key Laboratory of Carcinogenesis and Translational Research (Ministry of Education/Beijing) Peking University Cancer Hospital & Institute Beijing China

**Keywords:** deep learning, diffusion‐weighted imaging, rectal cancer, segmentation, U‐Net

## Abstract

**Purpose:**

Manual delineation of a rectal tumor on a volumetric image is time‐consuming and subjective. Deep learning has been used to segment rectal tumors automatically on T2‐weighted images, but automatic segmentation on diffusion‐weighted imaging is challenged by noise, artifact, and low resolution. In this study, a volumetric U‐shaped neural network (U‐Net) is proposed to automatically segment rectal tumors on diffusion‐weighted images.

**Methods:**

Three hundred patients of locally advanced rectal cancer were enrolled in this study and divided into a training group, a validation group, and a test group. The region of rectal tumor was delineated on the diffusion‐weighted images by experienced radiologists as the ground truth. A U‐Net was designed with a volumetric input of the diffusion‐weighted images and an output of segmentation with the same size. A semi‐automatic segmentation method was used for comparison by manually choosing a threshold of gray level and automatically selecting the largest connected region. Dice similarity coefficient (DSC) was calculated to evaluate the methods.

**Results:**

On the test group, deep learning method (DSC = 0.675 ± 0.144, median DSC is 0.702, maximum DSC is 0.893, and minimum DSC is 0.297) showed higher segmentation accuracy than the semi‐automatic method (DSC = 0.614 ± 0.225, median DSC is 0.685, maximum DSC is 0.869, and minimum DSC is 0.047). Paired *t*‐test shows significant difference (*T* = 2.160, *p* = 0.035) in DSC between the deep learning method and the semi‐automatic method in the test group.

**Conclusion:**

Volumetric U‐Net can automatically segment rectal tumor region on DWI images of locally advanced rectal cancer.

## 1 INTRODUCTION

Magnetic resonance imaging (MRI) is recommended by NCCN for the diagnosis and treatment of rectal cancer.^1^ Particularly, diffusion‐weighted imaging (DWI) may evaluate the microenvironment of tumor functionally and has become an indispensable imaging modality in addition to T2‐weighted imaging (T2WI). Many studies have shown that the apparent diffusion coefficients (ADC) value of a region of interest (ROI) inside rectal tumor may predict the response to chemoradiotherapy.^2^, ^3^ In addition, several prediction models have been established on DWI or ADC images by texture analysis, radiomics, or deep learning methods.^4^, ^5^, ^6^, ^7^, ^8^ The major limitation of these ROI‐based method is the requirement of manual segmentation. It takes 1–18.5 min to delineate a rectal tumor in pre‐treatment DWI images, considerably laborious and time‐consuming.^9^ Therefore, automatic segmentation of rectal cancer is needed as it may facilitate the construction of models for quantitative analysis.

The initial approach to automatic segmentation is based on level‐set by integrating different types of regularization into a problem of minimization, but these methods are bound to depend on manual intervention such as contour initiation or seed points.^10^ Recently, convolutional neural networks, particularly U‐shape networks (U‐Net),^11^ have been successfully employed in the fully automatic segmentation of medical images. Most of the rectal or colorectal tumor segmentation use CT or T2WI due to the high resolution, high contrast, and high signal‐to‐noise ratio.^12^, ^13^, ^14^, ^15^, ^16^, ^17^, ^18^, ^19^, ^20^, ^21^, ^22^, ^23^ Segmentation on DWI or ADC is rarely reported. DWI images suffer from noise and artifacts that may lead to false positives during segmentation. Although it is possible to copy the ROI from one pulse sequence (e.g., T2WI) to another one (e.g., DWI), sometimes the two sets of images are not well aligned due to body motion or involuntary bowel movement in the scanning interval. Therefore, automatic segmentation of rectal tumor on DWI is also necessary. Trebeschi et al. have proposed a network for rectal tumor segmentation by incorporating a fusion between T2WI and DWI.^24^ Deformable registration is required to align the two sets of images. If segmentation could be performed using only DWI data, it may avoid the possible error during registration.

In this work, a deep learning model is proposed for fully automatic segmentation of rectal tumors on DWI images. Instead of breaking the images into two‐dimensional (2D) slices or patches, a three‐dimensional (3D) volumetric U‐Net is constructed to utilize the spatial features in all three directions. The strategy suppresses false positive signals and avoids the need of following region selection. A semi‐automatic segmentation method of gray‐level thresholding was used for comparison to validate the advantage of using deep learning for segmentation.

## 2 MATERIALS AND METHODS

### 2.1 Participants

Patients were enrolled in this study with following inclusion criteria: (1) locally advanced rectal cancer confirmed by MRI and biopsy; and (2) MRI scanned at the same scanner with the same parameters. Exclusion criteria were: (1) lack of DWI images; and (2) insufficient image quality for measurement. Totally 300 patients were enrolled in this study.

### 2.2 MRI scanning

All participants were scanned at a 3.0 T MRI scanner (MR750; GE Healthcare) with T2WI, T1WI, DWI, and contrast‐enhanced T1WI pulse sequences. Only DWI data were analyzed in this study. The scanning parameters are listed in Table 1.

**TABLE 1:** Magnetic resonance imaging scanning parameters.

TR (s)	TE (s)	FOV (mm)	*b*‐value (s/mm^2^)	Matrix	Thickness (mm)	Gap (mm)
2.8	0.066	340	1000	256 × 256	4.0	1.0

Abbreviations: FOV, field of view; TE, time of echo; TR, time of repetition

### 2.3 Manual segmentation

Manual segmentation was used as the ground truth in this study. All manual segmentations were performed by two radiologists with 10 years experience of diagnosis of rectal cancer. Segmentation file was created by ITK‐SNAP software (www.itksnap.org)^25^ with a graphics tablet. Freehand delineation was performed on DWI images (*b* = 1000 sec/mm^2^). Tumors show high signals in DWI images scanned at large *b*‐value. Images of other pulse sequences (T2WI, T1WI, and dynamic contrast‐enhanced T1WI) were used as a reference.

### 2.4 U‐Net and data pre‐processing

**FIGURE 1:**
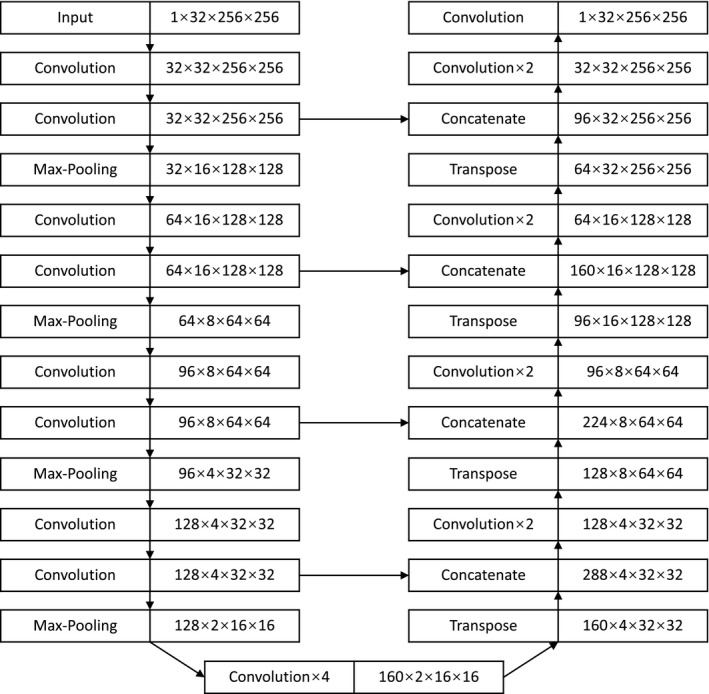
The structure of U‐Net for segmentation

The architecture of the networks is a U‐Net depicted in Figure 1. It is composed by 21 convolution layers with a kernel of 3 × 3 × 3, four max‐pooling layers (down‐sampling) with a kernel of 2 × 2 × 2, four transpose layers (up‐sampling) with a kernel of 2 × 2 × 2, four concatenate layers. The final layer uses a Softmax function to produce a probability between 0 and 1. Since rectal tumor was located at the central region of the imaging field during scanning, all images and segmentations were cropped or zero‐padded to the size of 256 × 256 × 32. After four repetitions of convolution and max‐pooling layers, the image was reduced to the size of 16 × 16 × 2. After four repetitions of convolution and transpose layers, the image was restored to the size of 256 × 256 × 32. The concatenate layers shortcut the images at the same depth. The total number of parameters in the U‐Net is 6,832,321. The network was designed and trained with TensorFlow (Version 1.4.0), Keras (Version 2.1.5) on the platform of Python (Version 3.6).

### 2.5 Training, validation, and test

All 300 patients were randomly divided into a training group (*n* = 180), a validation group (*n* = 60), and a test group (*n* = 60). Dice similarity coefficient (DSC) was used for training by defining 1‐DSC as the loss function. DSC is defined by Equation (1), where *V*(*A*) is the volume of the delineated tumor region (ground truth) and *V*(*B*) is the volume of automatic or semi‐automatic segmentation.

(1) $$\text{DSC}\left(A,\;B\right)=\frac{2V(A\cap B)}{V(A)+V(B)}$$

The validation group was used to optimize the hyperparameters such as learning rate, decay rate, and epochs by maximizing DSC. The test group was used to evaluate the network with DSC and Hausdorff distance (HD). HD is defined by Equation (2), where *d*(*a*, *b*) is the distance between point *a* and point *b*.

(2) $$\text{HD}\left(A,\;B\right)=\text{max}\left({\text{max}}_{a\in A}\left({\text{min}}_{b\in B}d\left(a,\;b\right)\right),{\text{max}}_{b\in B}\left({\text{min}}_{a\in A}d\left(b,\;a\right)\right)\right)$$

### 2.6 Semi‐automatic method

Semi‐automatic segmentation was performed by three steps: (1) the lower limit of the gray level inside the tumor region was manually assigned; (2) the regions above the threshold were automatically segmented; and (3) the largest connected region was automatically selected. This algorithm was designed based on an assumption that the rectal tumor is the largest connected region showing high signals in the DWI volumetric image.

## 3 RESULTS

The characteristics of the subjects in the training, validation, and test groups were summarized in Table 2. Continual features such as age and tumor volume were compared by ANOVA. Categorical features such as gender and clinical T‐stage were compared by Chi‐square method. There is no significant difference among the three groups considering the age, gender, clinical T‐stage, and tumor volume.

**TABLE 2:** Demographic and clinical characteristics of subjects in the training (*n* = 180), validation (*n* = 60), and test groups (*n* = 60)

Characteristics	Training group	Validation group	Test group	Statistics
Age (year) (mean ± SD)	57.2 ± 10.2	56.1 ± 9.4	54.6 ± 11.8	*F* = 1.462, *p* = 0.233
Gender (%)				
Male	113 (62.8)	39 (65.0)	41 (68.3)	*Χ*^2^ = 0.620, *p* = 0.734
Female	67 (37.2)	21 (35.0)	19 (31.7)	
Clinical T stage (%)				
T2a	29 (16.1)	9 (15.0)	5 (8.3)	*χ*^2^ = 19.96, *p* = 0.068
T2b	21 (11.7)	5 (8.3)	13 (21.7)	
T3a	58 (32.2)	23 (38.3)	12 (20.0)	
T3b	47 (26.1)	16 (26.7)	18 (30.0)	
T3c	7 (3.9)	0 (0)	3 (5.0)	
T4a	5 (2.8)	5 (8.3)	6 (10.0)	
T4b	13 (7.2)	2 (3.3)	3 (5.0)	
Tumor volume (voxel) (mean ± SD)	2160 ± 1888	2700 ± 2653	2564 ± 2357	*F* = 1.788, *p* = 0.169

**FIGURE 2:**
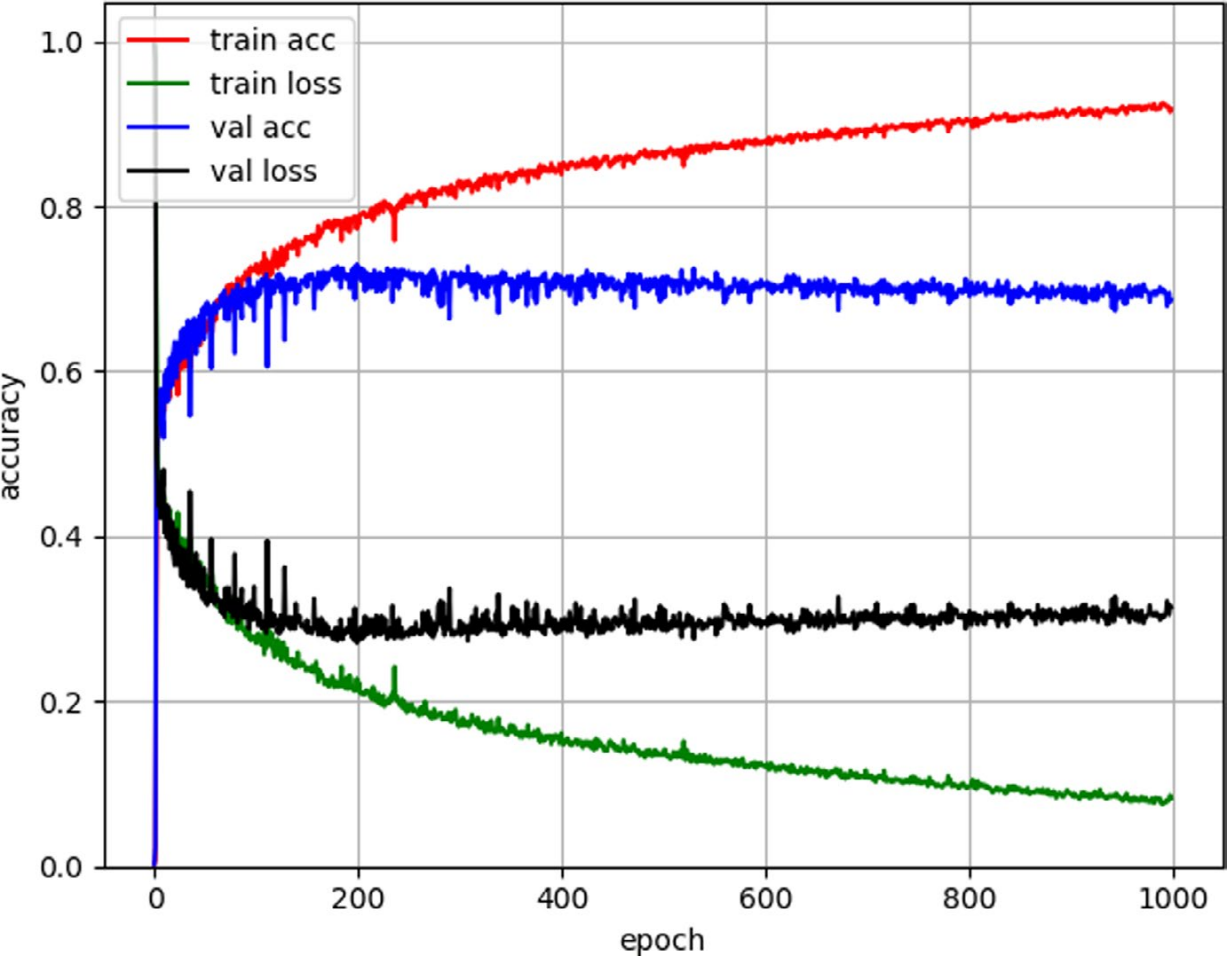
The training process of deep learning model. The accuracy is the mean dice similarity coefficient (DSC) value. The loss is 1‐DSC. The maximum accuracy appears at around 200 epochs

Optimal learning rate was set to 1e–4 and the decay rate was set to 1e–5. The training process is visualized in Figure 2. It shows the training accuracy, training loss, validation accuracy, validation loss from epoch 1 to 1000 epochs, where accuracy is the mean DSC. Accuracy reaches the maximum value at 200 epochs and decline. The network trained after 200 epochs was used for testing. DSC and HD were summarized in Table 3. On the test group, the mean DSC and median DSC of deep learning method are 0.675 and 0.702. The correlation between DSC and tumor volume is *R* = 0.371 (*p* = 0.004). It suggests that segmentation performs better at larger tumors than smaller tumors. Despite of manual intervention, semi‐automatic segmentation produces smaller DSC (mean is 0.614 and median is 0.685). Paired *t*‐test shows significant difference (*T* = 2.160, *p* = 0.035) in DSC between two methods.

**TABLE 3:** Dice similarity coefficient (DSC) and Hausdorff distance (HD) of the training, validation, and test groups

Methods	Semi‐automatic			Deep learning		
Groups	Training	Validation	Test	Training	Validation	Test
DSC mean	0.565	0.622	0.614	0.764	0.662	0.675
DSC std	0.249	0.209	0.225	0.087	0.147	0.144
DSC median	0.655	0.673	0.685	0.767	0.684	0.702
DSC max	0.867	0.904	0.869	0.910	0.886	0.893
DSC min	0.002	0.009	0.047	0.420	0.229	0.297
HD mean	22.596	19.196	25.069	10.936	16.169	15.891
HD std	16.721	11.081	18.615	6.706	8.894	10.041

**FIGURE 3:**
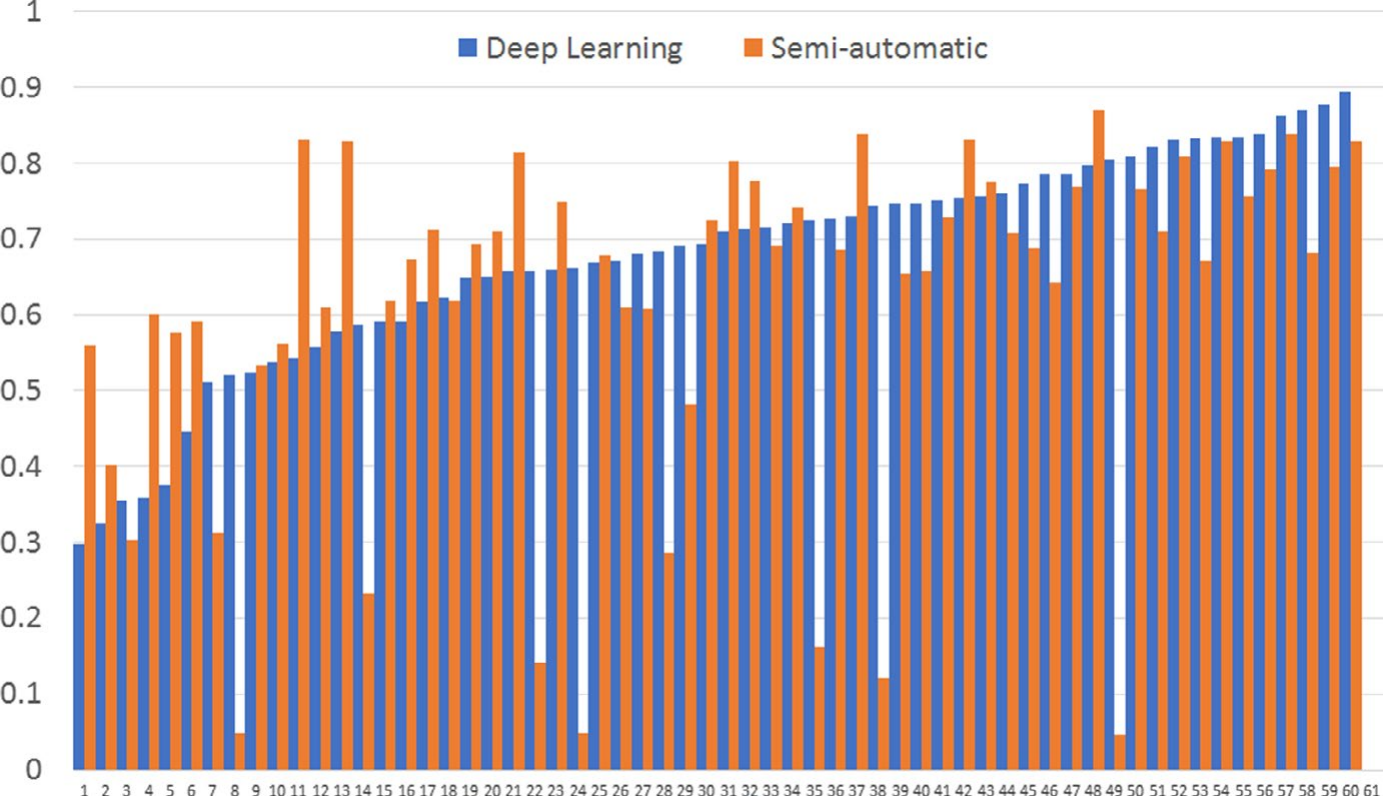
Dice similarity coefficient (DSC) of segmentation on each subject in the test group by the deep learning method and the semi‐automatic method

The DSC of each patient in test group was plotted in Figure 3, where the DSC of deep learning is arranged in an ascending order. Neighboring bars with blue and orange colors belong to the same subject. In general, the subjects producing low DSC by deep learning segmentation also produce low DSC by semi‐automatic segmentation, but the semi‐automatic segmentation show several cases with near‐zero DSC.

**FIGURE 4:**
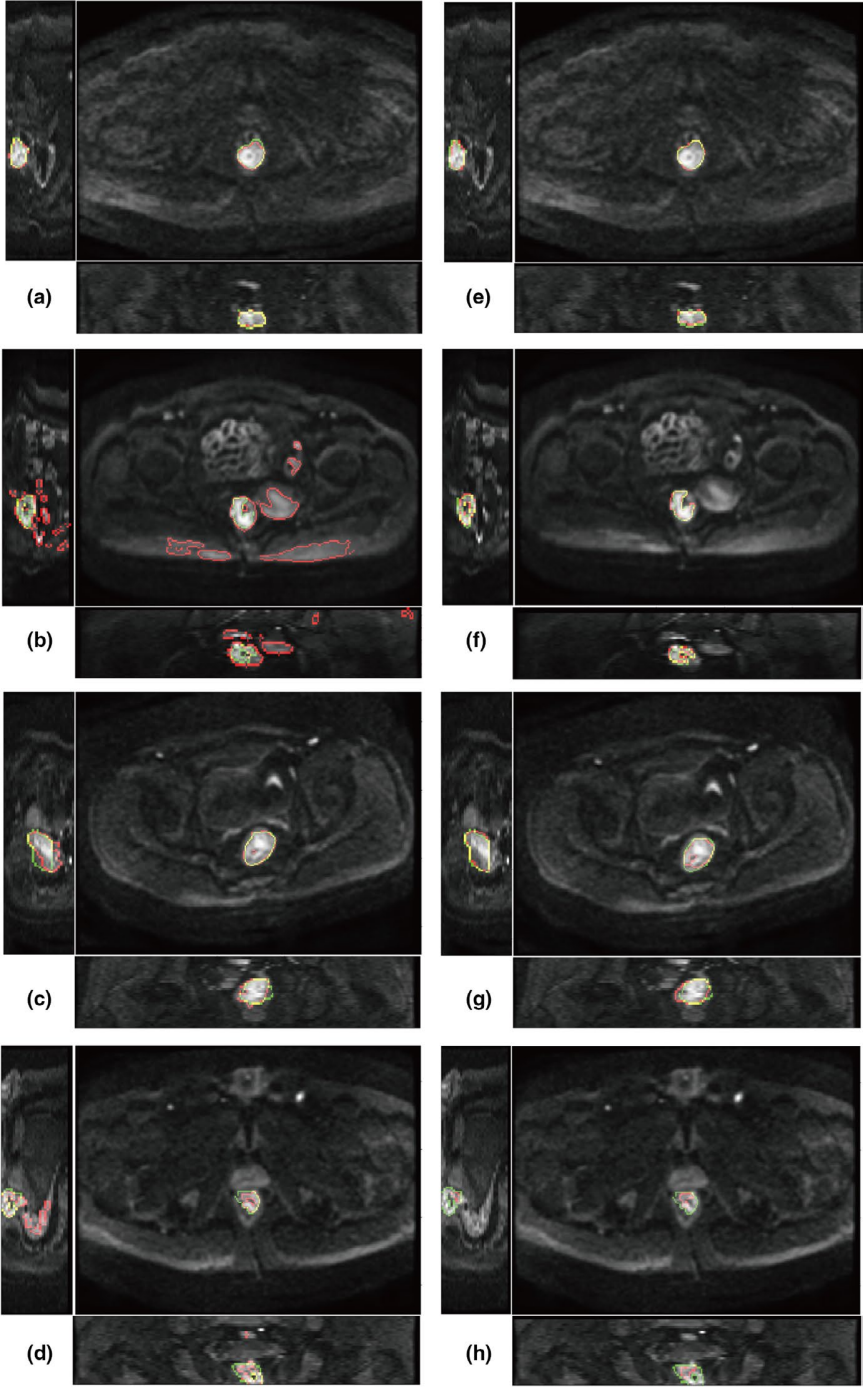
Example of segmentation. (a–b) semi‐automatic segmentation; (e–h) deep learning segmentation. The two images in each row are from the same patient. Green color shows the contour of ground truth delineated by radiologists. Red color shows the contour of segmentation. Yellow color is the overlap of green and red contours

Examples of segmentation were demonstrated in Figure 4 from three directions. The green contours are ground truth and the red contours are segmentation. The yellow contours are the overlap of green contours and red contours. Figure 4a–d was segmented by the semi‐automatic method. Figure 4e–h was segmented by the deep learning method. Each pair of images (a and e, b and f, c and g, and d and h) belongs to the same subject. The largest and the smallest DSC by semi‐automatic method are A (AUC = 0.869) and B (AUC = 0.047). The corresponding deep learning results are E (DSC = 0.798) and F (DSC = 0.805). The largest and the smallest DSC by deep learning method are G (DSC = 0.893) and H (DSC = 0.297). The corresponding semi‐automatic results are C (DSC = 0.830) and D (DSC = 0.559). Results shows that the semi‐automatic method tends to include more false positives if the gray level of adjacent tissues is close to the tumor region.

## 4 DISCUSSION

Region of interest segmentation has become a monotonous and time‐consuming task for radiologists since a huge number of delineated samples are needed for machine learning or deep learning. Automatic segmentation of tumor regions may free radiologists from manual delineation. Compared with most level‐set methods that need manual intervention, deep learning methods manage to achieve fully automatic segmentation. T2WI and DWI are the most useful MRI protocols for the diagnosis of rectal tumors. Several deep learning models have been established based on T2WI images, but segmentation on DWI images is rarely reported. Segmentation on each pulse sequence is necessary because the images may not keep aligned during the scanning of all pulse sequences. For example, if body motion or involuntary bowel movement happens during the interval between T2WI and DWI protocols, ROI delineated on T2WI data cannot be shared to DWI data. Trebeschi et al. have constructed a deep learning model to segment rectal tumor by a fusion of DWI and T2WI and managed to produce a DSC value of 0.70 and 0.68.^24^ Our model aimed to perform segmentation using DWI data alone. It avoids the potential errors in registration, especially when the signals and positions of pelvic normal structures are altered due to tumor growth.

Several network architectures have been proposed for segmentation, which are summarized in Table 4. Some studies use asymmetrical encoding–decoding, such as VGG‐like net for encoding and interpolation for decoding.^12^, ^17^ The most widely used net architecture is U‐Net, a symmetrical encoding–decoding structure.^11^ The encoding part down‐samples the image and the decoding part up‐samples the image. 2D U‐Net is commonly used due to the limitation of memory or computation time.^14^, ^18^ But 2D U‐Net may lose the spatial context along the slice direction of MRI data. In clinical practice, radiologists generally need to view multiple slices to identify a tumor according to its 3D structure. Analogically, 3D U‐Net has been applied on rectal tumor for volume‐to‐volume segmentation.^16^, ^20^ In this study, 3D U‐Net was used to convert a volumetric DWI image into a 3D probability map with the same size. The abundant 3D information may reduce false positives caused by noise or artifact. Results show that most of the cases generate a single connected region by thresholding probability at 0.5. Therefore, there is no need of implementing an additional step to select the largest connected region. For the methods that require region selection, if the largest connected region is not the target tumor, it will give a quite small DSC value even zero DSC just like the two examples of DSC = 0 given in the related work.^24^

**TABLE 4:** Related works on rectal tumor segmentation by deep learning

Author	Year	Modality	Network	Subjects (*n*)	Mean DSC
Trebeschi et al^24^	2017	T2WI+DWI	2D patch CNN	70	0.68, 0.70
Men et al^12^	2017	CT	2D DDCNN	278	0.88
Wang et al^14^	2018	T2WI	2D U‐Net	93	0.74 ± 0.14
Jian et al^15^	2018	T2WI	2D VGG‐16	512	0.84 ± 0.11
Soomro et al^16^	2018	T2WI	3D FCN	70	0.94
Men et al^17^	2018	T2WI, CT	2D CAC–SPP	70, 100	0.78, 0.85
Wei et al^18^	2019	CT	2D FCN U‐Net	107	0.81
Wang et al^19^	2019	T2WI	2D ResNet‐50	568	0.82
Soomro et al^20^	2019	T2WI	3D MSDNet	43	0.86 ± 0.02
Liu et al^21^	2019	CT	2D GAN	223	0.92 ± 0.01
Lee et al^22^	2019	T2WI	2D U‐Net	457	0.74 ± 0.19
Shi et al^23^	2020	CT	SG‐Unet	108	0.91,0.85

Abbreviations: CAC, cascaded atrous convolution; DDCNN, deep dilated convolutional neural network; FCN, fully convolutional neural networks; GAN, generative adversarial networks; MSDNet, multiscale dense networks; SG‐Unet, stacked generalization U‐shape networks; SPP, spatial pyramid pooling; T2WI, T2‐weighted imaging; U‐Net, U‐shape networks; VGG, visual geometry group.

In this study, a semi‐automatic method was used to compare with the proposed deep learning method. The semi‐automatic method requires manually assigning a thresholding value for voxel selection first and then automatically segment the largest connected region as the tumor region. The algorithm was designed based on two assumptions. First, it assumes that rectal tumor generally shows the highest signals in DWI images. Second, it assumes that tumor region is the largest connected region and the false positives are scattered smaller regions. Results show that the semi‐automatic method performs well at most subjects. However, several subjects produce quite small DSC depicted in Figure 3. The reason can be demonstrated by Figure 4b where the DWI signals at rectal tumor were too low to be discriminated from the surrounding structures. In contrast, deep learning segmentation did not produce such small DSC. Because deep learning can extract high‐level features by multiple convolutional layers, it may recognize the difference between rectal tumor and the surrounding structures.

The study is from a single center and all the subjects were scanned at the same MRI scanner with the same protocols and parameters, which is a major limitation of this study. Single data source makes it easy for training but difficult for general application. If data from multiple scanners were used, image normalization is required to minimize the difference in scaling and resolution. However, image normalization is still a difficult question for MRI because MRI signals are nonlinear with physical values and scanning parameters. Compared with DWI signals, ADC is an inherent MRI value of the tissue and less affected by scanning conditions. Therefore, segmentation on ADC map might be appropriate for multi‐center studies.

## 5 CONCLUSION

Our results demonstrate that the U‐Net model can perform accurate segmentation of rectal tumor on DWI images in most cases of locally advanced rectal cancer. Deep learning is a promising tool for fully automatic segmentation to overcome the obstacle of time‐consuming manual delineation.

## CONFLICT OF INTEREST

None.

## AUTHOR CONTRIBUTION

Hai‐Tao Zhu, Xiao‐Yan Zhang, and Ying‐Shi Sun were involved in designing this study. The neural network was designed by Hai‐Tao Zhu. Xiao‐Yan Zhang and Yan‐Jie Shi were involved in collecting the data and manual delineation. Statistical analysis was performed by Xiao‐Ting Li. The manuscript was drafted by Hai‐Tao Zhu and Xiao‐Yan Zhang.

## Data Availability

The data that support the findings of this study are available upon request from the corresponding author. The data are not publicly available due to privacy or ethical restrictions.
